# Unconventional Approaches to Prepare Triazine-Based Liquid Crystal Dendrimers

**DOI:** 10.3390/nano11082112

**Published:** 2021-08-19

**Authors:** Yao-Chih Lu, Hsiu-Fu Hsu, Long-Li Lai

**Affiliations:** 1 Department of Applied Chemistry, National Chi Nan University, No. 1 University Rd., Puli, Nantou 545, Taiwan; s103324901@mail1.ncnu.edu.tw; 2 Department of Chemistry, Tamkang University, No. 151, Yingzhuan Rd., New Taipei City 251, Taiwan; hhsu@mail.tku.edu.tw

**Keywords:** triazine, dendrimer, liquid crystal

## Abstract

Most triazine-based liquid crystalline (LC) dendrimers reported thus far are the main-chain LC macromolecules with long flexible chains at their periphery and attached to internal rigid or semi-rigid frameworks. Their formation of mesogenic phases often depends on the intermolecular face-to-face π–π interactions between dendritic molecules, which are unusual. Their mesogenic phases can also be formed by incorporation of mesogenic units to the dendritic skeletons through long flexible chains, as most side-chain LC dendrimers, in which the peripheral mesogenic units generally play the important roles. For main-chain triazine-based dendrimers, their morphology is maintained by restricted freedom of rigid or semi-rigid connecting units, and their formations of LC phases are therefore not straightforward to be controlled. In this review, we thus describe modulating of the intermolecular face-to-face π–π interactions between the triazine-based dendritic molecules, with the aim of forming LC phases through molecular design.

## 1. Introduction

**Scheme 1:**
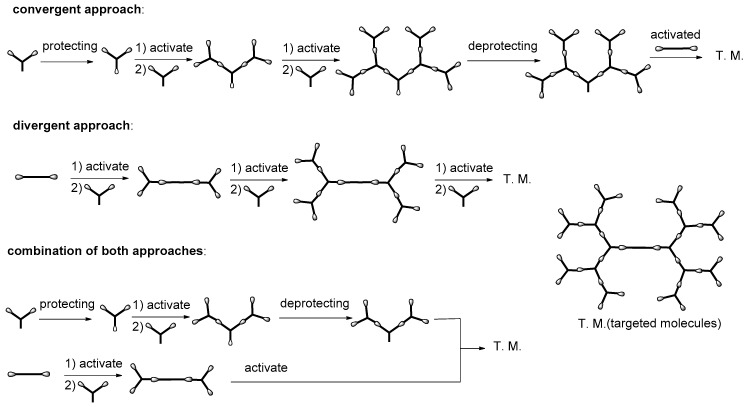
Preparation of dendrimers by the convergent, divergent, and combined approaches.

Since the discovery of dendrimers in the late 1970s, [1] there has been substantial development in their synthesis and study of their physical properties. Dendrimers generally consist of a central core, linking bridges and peripheral groups. They can be efficiently prepared by iterative processes through convergent, divergent, and combined approaches as demonstrated in Scheme 1 [2]. Although dendrimers may have a branched architecture and three-dimensional spatial arrangement, their morphology is still predictable. Formed by controllable step-by-step and iterative processes, dendrimers are a class of macromolecules with several characteristics that polymers do not have, such as monodispersity, controllable sizes, and internal cavities [2]. In particular, various types of functionally designed groups can be attached at the periphery of dendrimers to give desired properties for this type of macromolecule. Therefore, dendrimers have been extensively investigated and are currently applied in various fields, including drug delivery [3,4,5,6,7,8,9], catalysis [10,11,12,13,14,15,16], light harvesting [17,18,19,20,21,22], and porous materials [23,24,25,26,27].

With mesogenic units in the dendritic framework, dendrimers have also been observed to exhibit liquid crystal (LC) behaviors on thermal treatment. Compared with traditional LC usually with low molecular weight, LC dendrimers often have a higher molecular weight and exhibit columnar mesophases on thermal treatment and are 2-D regular materials, thus yielding better self-assembling ability in a long-range domain and which have usefulness as solvating candidates in opto-electronic devices [28,29,30,31,32,33,34]. LC dendrimers can generally be divided into side-chain dendrimers in which the mesogenic units are incorporated at the periphery of molecules and main-chain dendrimers in which the mesogenic moieties are designed as linking moieties within the internal framework. Most LC dendrimers are side-chain LCs with flexible cores or linking units and peripheral mesogenic units linked to the internal dendritic structures through long flexible chains. The mesogenic units are important in inducing their LC behavior, and this type of LC dendrimer mostly consists of PMAMA (polyamidoamine), PPI (polypropylimine), Si-derivatives (siloxanes, carbosilanes, and carbosilazanes), and polyester as their dendritic network, all of which have been reviewed extensively [2,35,36,37,38]. Special functional moieties, such as fullerene and ferrocene, may be incorporated in side-chain LC dendrimers for particular properties as reported in literatures [2,36,39]. There are fewer main-chain LC dendrimers [40,41,42,43,44,45] than side-chain LC dendrimers because mesogenic moieties are used as bridging units in their internal structures, leading to a rigid or semi-rigid dendritic framework in the main-chain LC dendrimers in which flexible alkyl chains are frequently grafted at the periphery to increase their solubility in organic solvents. Therefore, in contrast to side-chain LC dendrimers in which the LC phases are mostly induced by the peripheral mesogenic units, both the rigid or semi-rigid internal frameworks and flexible peripheral chains are all important in inducing mesophases for the main-chain LC dendrimers [2,36,37], and this leads the formation of mesophases of the main-chain LC dendrimers to be difficult to control. As in the rod-like liquid crystals, the ionic, H-bond, and metallo interactions are also extensively employed in LC dendrimers, which also consist of side-chain and main-chain types of LC materials, depending on whether their mesogenic units are incorporated in the internal framework or at the periphery of dendritic network, which have been reviewed in the literature [46,47,48].

**Scheme 2:**
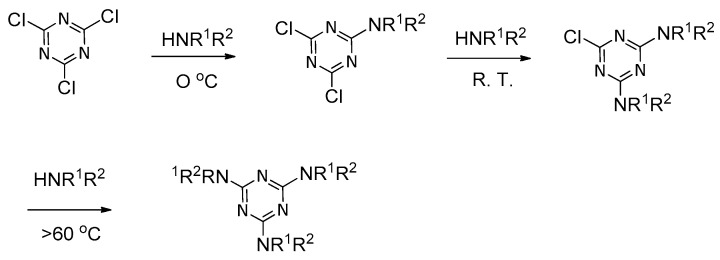
Reaction of amines with cyanuric chlorides at different temperatures.

Cyanuric chloride, a simple heterocycle with three reactive sites on its ring, can be used as a branching unit for efficiently preparing dendrimers. For instance, the first chloro substituent can be replaced by alkylamines at 0 °C, and subsequently, the second and the third substituents can undergo similar substitutions at room temperature and above 60 °C, respectively (Scheme 2) [49,50]. The triazine-based dendrimers were first prepared by Takagi [51] and later investigated by others [52,53]. Little progress on LC dendrimers based on the triazine unit has been reported since then. To the best of our knowledge, in 2008, Lai and co-workers were the first to report LC dendrimers on the basis of the 1,3,5-triamino-*s*-triazine unit [54]. Kotha and colleagues also prepared 1,3,5-triphenyl-*s*-triazine-based dendrimers, which exhibited a columnar phase after being mixed with 1:1 molar ratio of trinitrofluorenone [55]. However, there has been less study of triazine-based LC dendrimers, and most are main-chain types of LC dendrimers. Induction of the LC phases of triazine-based dendrimers can be achieved by manipulating the molecular morphology of the dendrimer themselves or by adding extra components to form an LC dendritic mixture by intermolecular H-bond or face-to-face π–π interactions. By reviewing this system, we hope to guide future work in this field.

## 2. Formation of Triazine-Based LC Dendrimers by Manipulating Their Molecular Morphology

### 2.1. LC Dendrimers with C_2_ Symmetry

**Figure 1:**
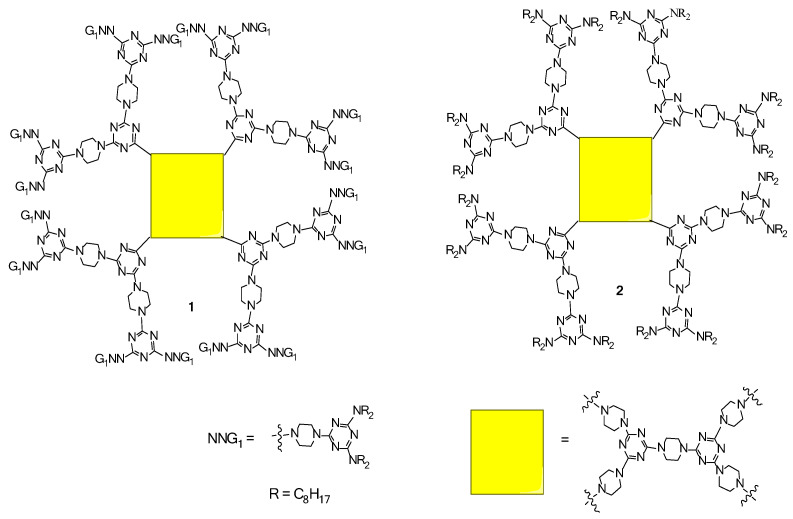
Structures of dendrimers (**1**) and (**2**) with C2-symmetry.

The LC dendrimer with a C2-symmetry on the basis of a 1,3,5-triamino-s-triazine unit was first reported by Lai and colleagues [54]. They prepared the fourth generation LC dendrimer **1** by the convergent approach (Figure 1), with a single molecular weight of ~12,479. Compound **1** is a monotropic material, showing no mesophase during heating but a columnar hexagonal phase between ~160 and ~143 °C on cooling. Additionally, compound 1 did not show any significant absorbance beyond 280 nm in the UV-vis spectrum and started to decompose at 300 °C under nitrogen by TGA analysis, which is an important characteristic required for opto- and electro-applications. However, the third generation triazine-based dendrimer **2** did not exhibit mesogenic behavior on thermal treatment, isotropic at ~133 °C on heating, and crystallized at ~98 °C on cooling. Computer simulations for the conformations of dendrimers **1** and **2** showed that the steric effect from the peripheral skeleton of **1** is more significant than that of **2** [54], i.e., the dendritic conformation of **1** is less coplanar due to congestion between the branched mainframe, thus reducing the face-to-face π–π interactions between dendritic molecules and forming the columnar phase on thermal treatment. Compared with traditional disk molecules, dendrimer **1** with a C2-symmetry having a columnar LC phase on cooling, is unusual. To exhibit columnar mesophases, the traditional C2-symmetry molecules may consist of a macrocyclic core [56,57]. Introducing a chiral core to result in helical stacking columns by modulating the intermolecular π–π interaction between molecules is another approach [58].

### 2.2. LC Dendrimers with C_3_ Symmetry

**Figure 2:**
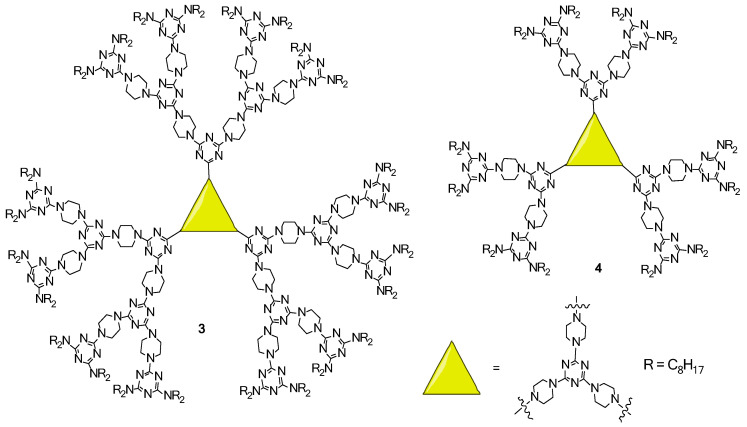
Structures of dendrimers (**3**) and (**4**) with C3-symmetry.

In addition, deforming the planarity of trigonal disk molecules to reduce face-to-face π–π interactions in traditional molecules can also lead to the formation of columnar mesophases [59]. Therefore, changing the shape of the rigid cores can be an efficient approach for preparing dendrimers with columnar LC phases, and thus two C3-symmetry dendrimers based on a triazine unit have been reported (Figure 2) [60]. Dendrimer **3**, with a molecular weight of ~9248, is a monotropic material, which did not exhibit any mesogenic behavior on heating but showed a columnar phase between ~137 and ~100 °C on cooling. Dendrimer **4** exhibited a columnar phase from ~141 to ~173 °C on heating and from ~168 to ~136 °C on cooling.

As demonstrated by dendrimers **1** and **2**, high molecular weight deforms the planar mainframe of dendritic molecules and leads to the formation of LC phases on thermal treatment. However, the molecular weight of dendrimer **4** (~4423) is much smaller than that of dendrimer **2** (~6034). Apparently, the *C_2_*-to-*C_3_* approach significantly changes planarity of the internal mainframe of dendrimers. Thus **4** exhibits a columnar LC phase during the thermal process although there is less congestion from the dendritic mainframe when compared with that of **2**.

### 2.3. LC Dendrimers with the Loss of C_2_ Symmetry by Changing the Peripheral Groups

**Figure 3:**
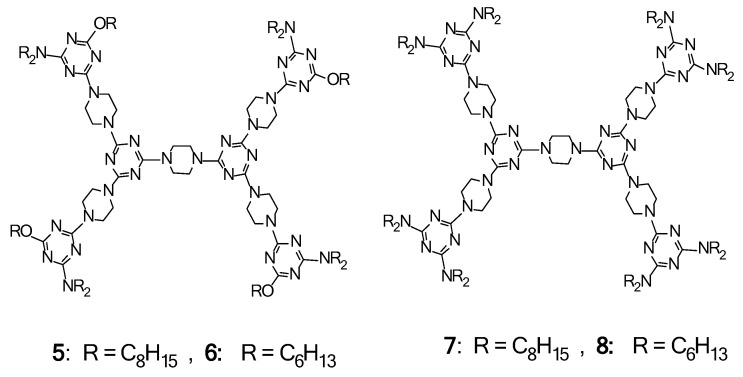
Structures of dendrimers (**5**) and (**6**) without a C2-symmetry and structures of dendrimers (**7**) and (**8**) with a C2-symmetry.

**Figure 4:**
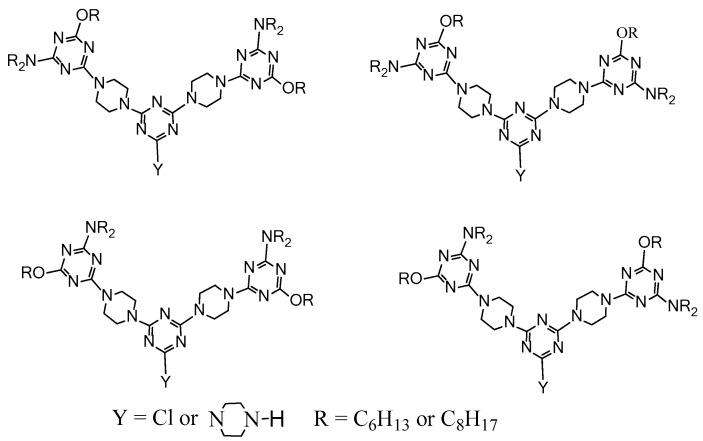
Structural isomers of dendrons to form dendrimers (**5**) and (**6**).

Reducing the intermolecular face-to-face π–π interactions between the molecules of triazine-based dendrimer seems to be a good strategy for forming mesogenic phases on thermal treatment. According to literature [61], mixing two pure components can reduce the molecular interaction in the solid state, and thus lowers the melting point of the mixture. For example, the m.p. of (R)-(−)-2-amino-1-propanol is ~24–26 °C, which is the same as that of (S)-(+)-2-amino-1-propanol, but the m.p. of the 1:1 mixture of these two components drops to ~8–10 °C. Accordingly, four triazine-based LC dendrimers were prepared (Figure 3) [62]. Dendrimer **5**, with the loss of the C2-symmetry, exhibited a columnar phase from ~95 to ~152 °C on heating and from ~147 to ~64 °C on cooling. Dendrimer **6**, also with the loss of the C2-symmetry, is a monotropic material, only showing a columnar phase between ~123 and ~145 °C on heating. Dendrimers **7** and **8**, with a C2-symmetry, did not exhibit any mesogenic phase on thermal treatment. Dendrimer **7** became isotropic at ~132 °C on heating and crystallized at ~117 °C on cooling; and dendrimer **8** became isotropic at ~229 °C on heating and solidified at ~221 °C on cooling. Dendrimers **5**, **6**, **7**, and **8** all have similar molecular weights of ~2368, ~2030, ~2813, and ~2363, respectively. Based on the molecular simulation investigations [62], they are almost co-planar and the congestions between their dendritic frameworks are negligible. However, dendrimers **5** and **6** are isomeric. As shown in Figure 4, dendrimers **5** or **6**, in principle, can be produced from the combination of the corresponding Cl-dendron and piperazino-dendron, respectively. Each dendron consists of at least four isomers, and therefore, dendrimers **5** and **6** have many isomers in their solid stackings, reducing their intermolecular face-to-face π–π interactions and forming LC phase during the thermal process.

### 2.4. LC Dendrimers with the Loss of C_2_ Symmetry by Changing the Central Cores

**Figure 5:**
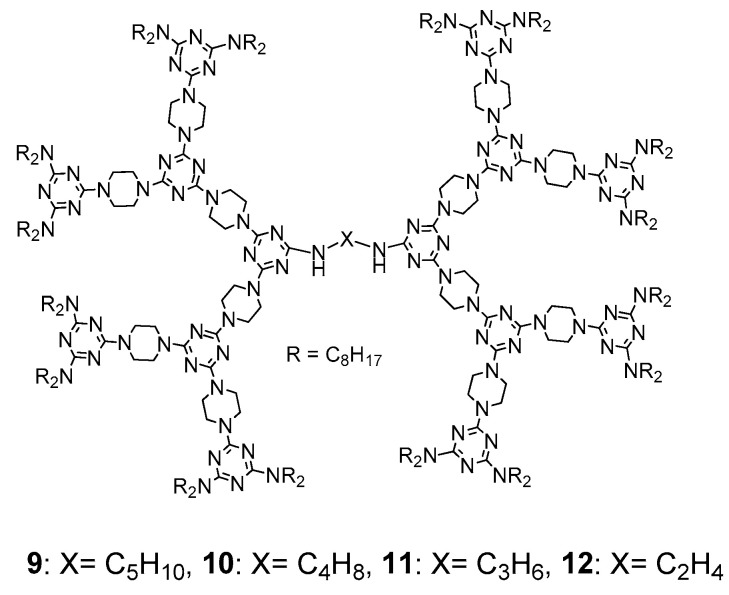
Structures of dendrimers (**9**–**12**).

Partially changing the peripheral group to break *C_2_*-symmetry of molecules can lead dendrimers to contain isomers, and face-to-face π–π interactions between dendritic molecules are thus modulated to form the LC phase with thermal treatment. This strategy can also be applied to central linkers of dendrimers. With the peripheral group fixed by the dialkylamino units, the central linker is modulated as C_5_H_10_, C_4_H_8_, C_3_H_6_, and C_2_H_4_ (Figure 5) [63]. Dendrimers **9** and **11**, with the odd-number carbon linker in the dendritic core, are LCs. Dendrimer **9** was observed to exhibit a columnar phase from ~109 to ~168 °C on heating and from ~146 to ~87 °C on cooling. Dendrimer **11** showed a columnar phase between ~107 and ~144 °C on heating and between ~115 and ~81 °C on cooling. Dendrimers **10** and **12** with the even-number carbon linker in the dendritic core are not liquid crystalline. The solid-phase-to-isotropic-phase (solid-to-iso) transition temperatures for dendrimers **10** and **12** are ~132 and ~147 °C, respectively, on heating, and the isotropic-phase-to-solid-phase (iso-to-solid) transition temperatures for **10** and **12** are ~100 and ~90 °C, respectively, on cooling.

**Figure 6:**
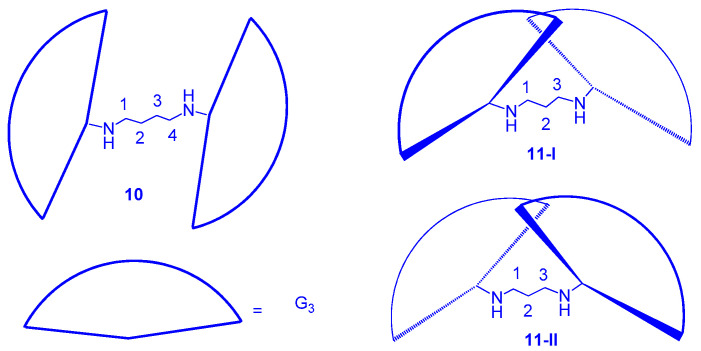
Isomers of dendrimers (**10**) and (**11**).

Molecular weights for dendrimers **9**–**12** are similar: ~6050, ~6036, ~6022, and ~6008, respectively. A computer simulation for molecular conformations of dendrimers **9** and **10** showed that **9** is asymmetrical and **10** is symmetrical along with the central linkers, and a diagram can show their differences [63]. As shown in Figure 6, dendrimer **10**, with a 4-carbon linker in the dendritic core, allows the dendronic halves to be located at the *anti*-position of the C_4_H_8_ linker to reduce repulsions between Hs (at C_2_ and C_3_) and the dendronic halves. Therefore, the molecular conformation of **10** is almost symmetrical. Accordingly, dendrimer **11** with a 3-carbon linker leads both the dendronic halves to exist in the *syn*-position of the C_3_H_6_ linker to reduce corresponding repulsions; in this conformation, two isomers of dendrimer **11** should exist since the left dendronic half may be on the top or bottom of the right dendronic half (Figure 6). Because of the isomeric effect, the intermolecular face-to-face π–π interactions are reduced in the solid stacking, and the columnar phases of dendrimers **9** and **11** are thus formed on thermal treatment. To further understand the isomeric effect on influencing their stackings in LC state, their molecular volumes (*V*_m_) and cell volumes (*V*_cell_) were calculated on the basis of powder-XRD data of dendrimers **9** and **11** [63]. Since the calculated volumes are temperature-dependent, and the XRD data for **9** and **11** were collected at different temperatures, the *V*_m_/*V*_cell_ ratios, instead of *V*_m_ or *V*_cell_, were compared to remove the temperature factor. The *V*_m_/*V*_cell_ ratios of **9** and **11** were calculated to be ~1.03 and ~0.95, respectively. For the non-mesogenic compounds **10** and **12**, crystalline XRD data were also used for calculation, assuming that both formed the columnar hexagonal crystalline phase; the *V*_m_/*V*_cell_ ratios of **10** and **12** were estimated to be ~0.92 and ~0.87, respectively.

The ratios of *V*_m_/*V*_cell_ for **9** and **11** are larger than those for **10** and **12**, indicating that the stackings of **9** and **11** in the solid state are somewhat looser than those of **10** and **12**. This further indicates that the isomeric effect on dendrimers can probably modulate the face-to-face π–π interactions between dendritic molecules.

**Figure 7:**
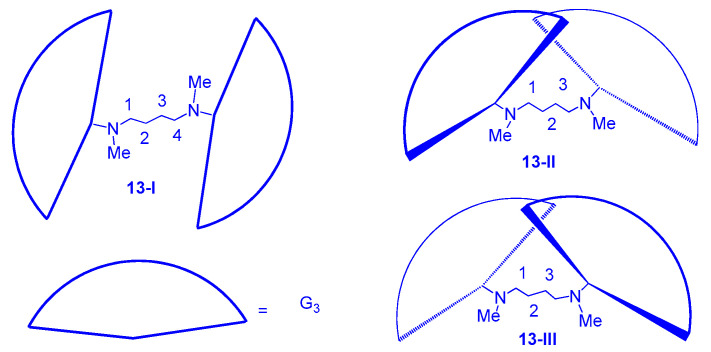
Isomers of dendrimers (**13**-**I**), (**13**-**II**), and (**13**-**III**).

**Figure 8:**
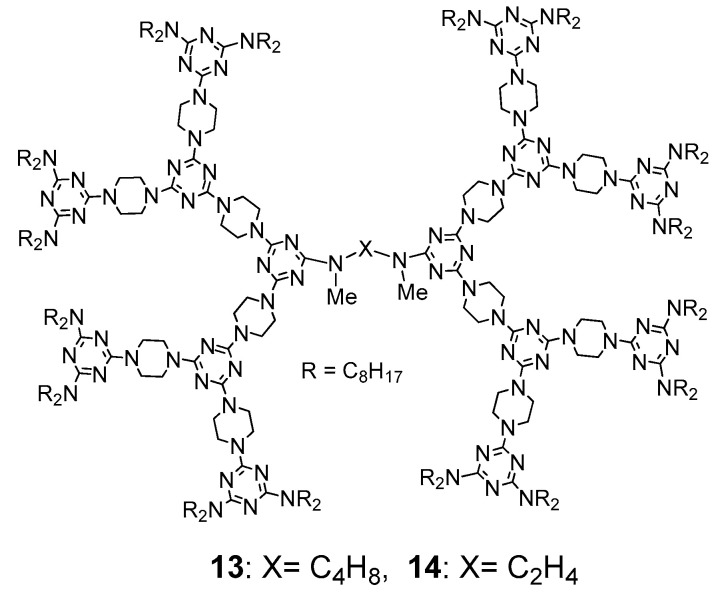
Structures of dendrimers (**13**) and (**14**).

As indicated previously, the repulsion between dendronic halves and the Hs (at C_2_ or C_3_) significantly influences the conformation of dendrimer **10**. If the central linker of **10** was methylated on nitrogen, then the repulsion between Me and the Hs (at C_2_ or C_3_) should increase, although the repulsion should be smaller than that between the dendronic halves and the corresponding Hs (at C_2_ or C_3_). However, this should convert several of the dendronic halves at the *anti*-position of C_4_H_8_ linker to the *syn*-position. Specifically, dendrimer **13** should have three isomers in the solid state, as shown in Figure 7 [64], although most of the **13** exists as the form of isomer **13-I** and, as expected, dendrimer **13** exhibited a columnar phase on thermal treatment (Figure 8). The mesogenic range is from ~111 to ~123 °C on heating and from ~115 to ~94 °C on cooling. Accordingly, dendrimer **14** has three isomers in the solid state but showed only a columnar phase between ~94 and ~72 °C on cooling without showing any mesogenic behavior on heating.

The solid-to-iso transition temperatures of dendrimers **10** and **12** on heating are ~132 and ~147 °C, respectively, which are higher than those of corresponding dendrimers **13** and **14** (~118 and 123 °C, respectively). This indicates that methylation of nitrogen in the central linker affects their molecular morphology and somewhat loosens their solid stacking. Based on the powder-XRD data of dendrimers **13** and **14**, their ratios of *V*_m_/*V*_cell_ were also calculated. The *V*_m_/*V*_cell_ ratio of **13** was ~0.92, similar to that of **10** (~0.92) and the *V*_m_/*V*_cell_ ratio of **14** was ~0.80, which is smaller to that of **12** (~0.87).

**Figure 9:**
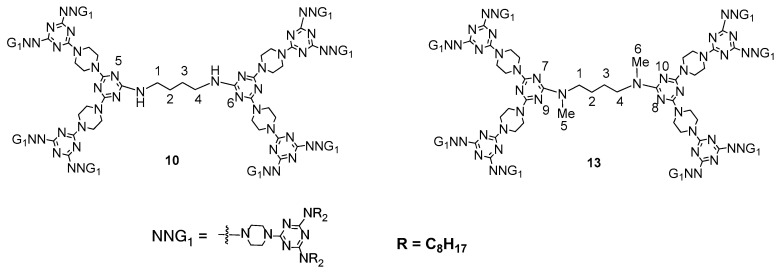
The numbering on carbons for indicating intramolecular H-bond interactions between H and N of dendrimers (**10**) and (**13**).

Although methylation of nitrogens in the central linker of dendrimers converts non-mesogenic **10** and **12** to become mesogenic **13** and **14**, respectively, the isomeric effect seems to not be significant in reducing intermolecular face-to-face π–π interactions, allowing dendrimers **13** and **14** to be looser in the solid stacking than dendrimers **10** and **12**. Based on the optimal conformation of **13** [64], it was discovered that four intramolecular H-bond interactions arise between N_a_ (a = 7, 8, 9, 10) and H (at C_b_; b = 1, 4, 5, 6), respectively, as shown in Figure 9. Accordingly, only two intramolecular H-bond interactions arise between N_a_ (a = 5, 6) and H (at C_b_; b = 1, 4,) in dendrimer **10**. Therefore, dendrimer **13** that mostly exists in the form of **13-I** should be more rigid and is less distorted than dendrimer **10**, and therefore the void space inside the solid stacking of **10** should be larger than that inside the dendritic framework of **13-I**. However, because of the isomeric effect, the face-to-face π–π interactions between dendritic molecules of **13** is reduced and therefore its dendritic framework is fluffier. As a result, dendrimers **10** and **13** have almost the same *V_m_*/*V_cell_* ratio (~0.92) on the basis of balancing the two conflicting factors. When the central linker is shortened, congestion from dendritic halves of **12** is more vigorous and the distortion allows dendrimer **12** to become more porous. Therefore, the *V_m_*/*V_cell_* ratio of **12** (~0.87) is larger than that of **14** (~0.80).

**Figure 10:**
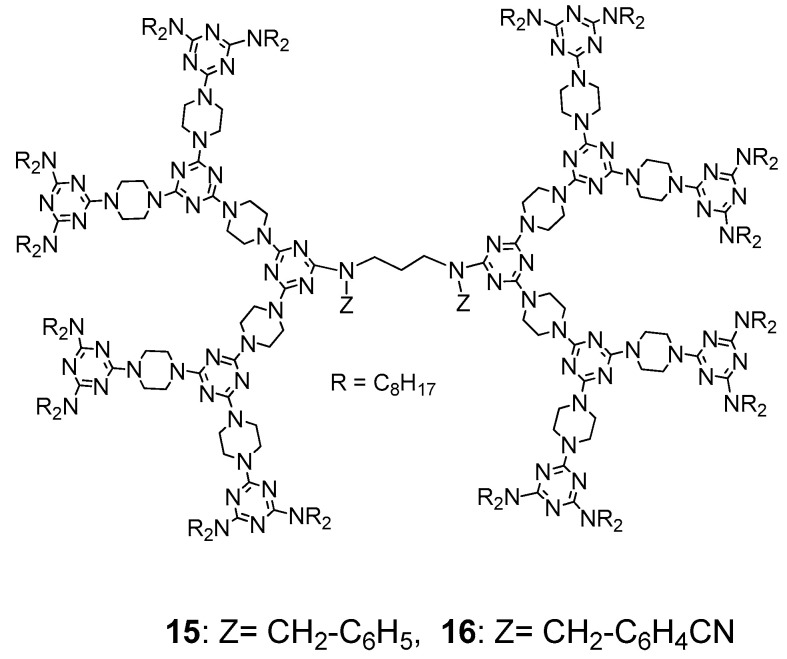
Structures of dendrimers (**15**) and (**16**).

The central linkers in triazine-based dendrimers significantly influence their morphology and consequently affect the formation of the mesophase during the thermal process. Instead of N-alkylation of an even-number carbon central linker, this strategy was also undertaken to an odd-number carbon central linker. As dendrimer **11** has already been observed to exhibit a columnar phase on thermal treatment, a strong CN polar group was incorporated in the central linker to investigate its influence on the mesogenic behavior. Dendrimers **15** and **16** were therefore further prepared for studying their LC properties (Figure 10) [65]. Dendrimer **15** exhibited a columnar phase between ~102 and ~118 °C on heating and between ~115 and ~82 °C on cooling. Dendrimer **16** showed a columnar phase from ~88 to ~144 °C on heating and from ~138 to ~68 °C on cooling. Compared with the DSC behaviors of **11**, the solid-to-iso transition temperature of **15** (~118 °C) was lower than that of **11** (~144 °C) on heating, indicating that the alkylation on N in the central linker increased the steric congestion of triazine-based dendrimer and somewhat loosened their solid stacking as shown in the study of dendrimers **10**, **12**, **13** and **14**. The solid-to-iso transition temperature of **16** (~144 °C) was similar to that of **11** (~144 °C), and the mesogenic range of **16** on cooling (from ~138 to ~68 °C) was broader than that of **11** (from ~115 to ~81 °C). The CN group in the central linker not only lowered the solidifying temperature but also raised the isotropic-phase-to-columnar-mesophase (iso-to-col) transition temperature on cooling. In principle, the steric congestion from benzyl moiety in the central linker of dendrimers **15** should not differ from and 4-cyanobenzyl moiety in **16**, but their mesogenic behaviors on thermal treatment are significantly different. The strong polarity of the CN group strengthens intermolecular interactions between face-to-face dendritic molecules. For further understanding, the *V_m_*/*V_cell_* ratios of **15** and **16** were estimated on the basis of their powder-XRD data as ~0.80 and ~0.98, respectively. The *V_m_*/*V_cell_* ratio of dendrimer **16** was larger than that of **11** (~0.95), but that of dendrimer **15** was smaller, indicating that the loose order of their stacking is **16** > **11** > **15**. As dendrimers **11**, **15**, and **16** all show a columnar phase in the mesogenic range, the face-to-face distances between two dendritic molecules (*d_slice_*) for **11**, **15**, and **16** can be obtained from the powder-XRD data in a similar manner and are calculated to be 13.14, 13.28, and 13.68 Å, respectively. The *V_m_*/*V_cell_* ratios and *d_slice_* distances show that the strong polarity of CN group significantly increased the face-to-face distance between dendritic molecules within columns. This reasonably explains that the col-to-solid transition temperature on cooling of **16** is lower than those of **11** and **15**. However, as indicated previously, the solid-to-iso transition temperature of **16** (~144 °C) is similar to that of **11** (~144 °C) on heating, which can also be ascribed to the strong polarity of the CN group. During the heating process, the strong intermolecular polar-to-polar interactions within columns led dendrimer **16** to have the same solid-to-iso transition temperature as **11**, although the stacking of **16** was looser. Neither dendrimer **11** nor **15** contains any functional group in the central linker, but the *d_slice_* distance of **15** within columns is 13.28 Å, only slightly larger than that of **11** (13.14 Å). The more void space between dendritic molecules of **15** is occupied by the benzyl group, which results in the lower *V_m_*/*V_cell_* ratio of **15** (0.80) versus that of **11** (0.95). Since the *d_slice_* distances of **11** and **15** do not differ from each other, the mesophase ranges of both dendrimer are also similar to each other.

## 3. Formation of Triazine-Based LC Dendrimers by Adding Extra Components

### 3.1. Formation of LC Dendrimers by Intermolecular π–π Interaction

**Figure 11:**
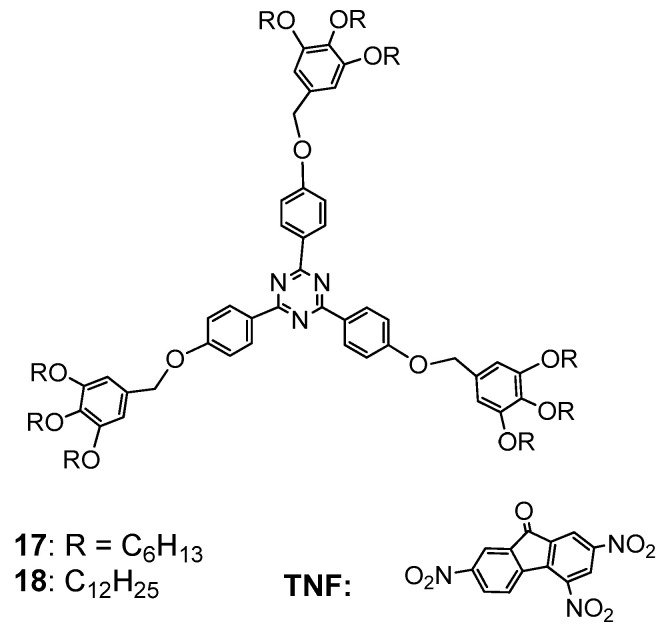
Structures of dendrimers (**17**) and (**18**).

In addition to manipulating the morphology of dendritic molecules to form their LC phase, mixing extra components to non-liquid-crystalline (non-LC) triazine-based dendrimers can lead to the mesophase formation. Dendrimers **17** and **18** were reported to be non-LC on thermal treatment (Figure 11) [55]. Compound **17** became isotropic at ~66 °C on heating but no transition was observed on cooling. Compound **18** became isotropic at ~57 °C on heating and crystallized at ~32 °C on cooling. After mixing with 1:1 molar ratio of trinitrofluorenone (**TNF**), the mixture of **17**-**TNF** showed a columnar phase between ~56 and ~78 °C on heating but still no transition was observed on cooling. The mixture of **18**-**TNF** exhibited a columnar phase from ~51 to ~102 °C on heating and from ~97 °C to room temperature on cooling [55]. It is believed that **TNF** forms a complex mixture with **17** or **18** through the face-to-face π-π interactions, thereby adjusting the distance between dendritic molecules and showing LC phases on thermal treatment.

### 3.2. Formation of LC Dendrimers by Intermolecular H-Bond Interaction

**Figure 12:**
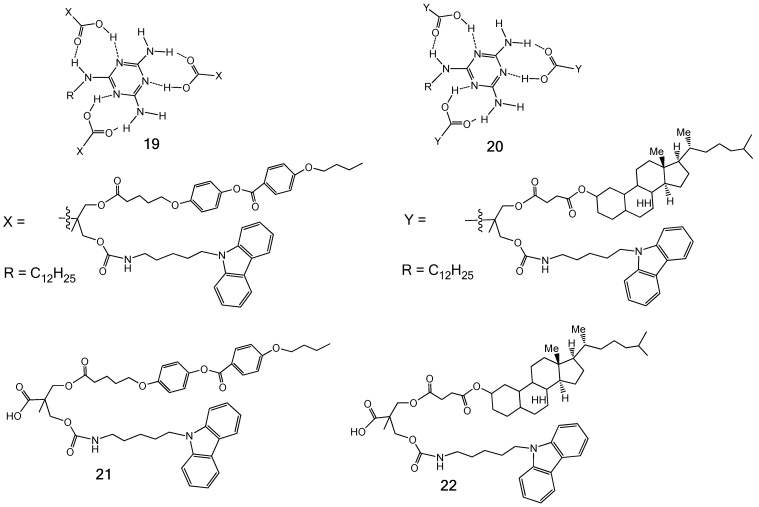
Structures of dendrimers (**19**) and (**20**) and and dendrons (**21**) and (**22**).

Apart from the face-to-face π–π interactions, the LC dendritic mixtures can also be formed by the intermolecular H-bond interaction. Mixing 2,4-diamino-6-dodecyl-1,3,4- triazine with carboxylic acid-containing dendron in a 1:3 ratio generates H-bonded dendrimers **19** and **20**, as shown in Figure 12 [66]. The acid-containing dendrons **21** and **22** did not exhibit any mesophase on thermal treatment. Compound **21** showed only a glass-phase-to-isotropic-phase (glass-to-iso) transition temperature at ~52 °C on heating, and compound **22** showed a glass-to-iso transition temperature at ~46 °C on heating. Instead of a columnar phase, dendrimer **19** exhibited a nematic phase between ~4 and ~35 °C on heating, and dendrimer **20** was observed to exhibit a SmA phase from ~43 and ~70 °C on heating. Neither dendrimers nor dendrons were reported to show any mesophase on cooling. A carbazole unit was incorporated in dendrimers and dendrons for studying their photoconductivities, but the performances of dendrons **21** and **22** were slightly better than those of dendrimers **19** and **20**, respectively.

## 4. Possible Applications

As shown in the literature, the triazine-based LC dendrimers are thermally stable and nearly transparent beyond 280 nm in the UV/Vis range [54]. Moreover, these dendrimers are electrochemically stable in solution and can survive in acidic media for a period of time [52]. These characteristics are important and are often required for opto- and electro-applications. In addition, LC dendrimers often have a high molecular weight, exhibiting columnar phases on thermal treatment and thus exhibiting good self-assembling ability in a long-range domain. In solvating guest materials, such as carbon nanotubes and nano-size catalysts, the LC dendrimers can lead the physical properties of guest molecules to be significantly displayed [28,29,30,31,32,33,34]. As shown in the introduction, dendrimers have been shown to be useful in the fields of catalysis [10,11,12,13,14,15,16] and light harvesting [17,18,19,20,21,22]. With the assistance of LC dendrimers, the guest molecules should be more regularly arranged, thus exhibiting their physical characteristics significantly.

## 5. Conclusions and Prospect

Three approaches have been described to prepare triazine-based dendrimers with mesogenic phase on thermal treatment. The first is to properly incorporate mesogenic units onto triazine and piperazine framework in a straightforward manner by molecular design.

The second approach is to mix components to form LC dendritic mixtures by adjusting intermolecular face-to-face π–π interactions, which is valuable for 1,3,5-triamino-*s*-triazine-based LC dendrimers. The mixing approach allows versatile preparation of 1,3,5-triamino-*s*-triazine-based dendrimers, which were observed to form strong π–π interactions with tetrafluorbenzoquinone (TFB) in CH_2_Cl_2,_ and the red shift of absorbance from the dendrimer-TFB complexes in the UV-Vis spectra can be up to ~180 nm [67].

**Figure 13:**
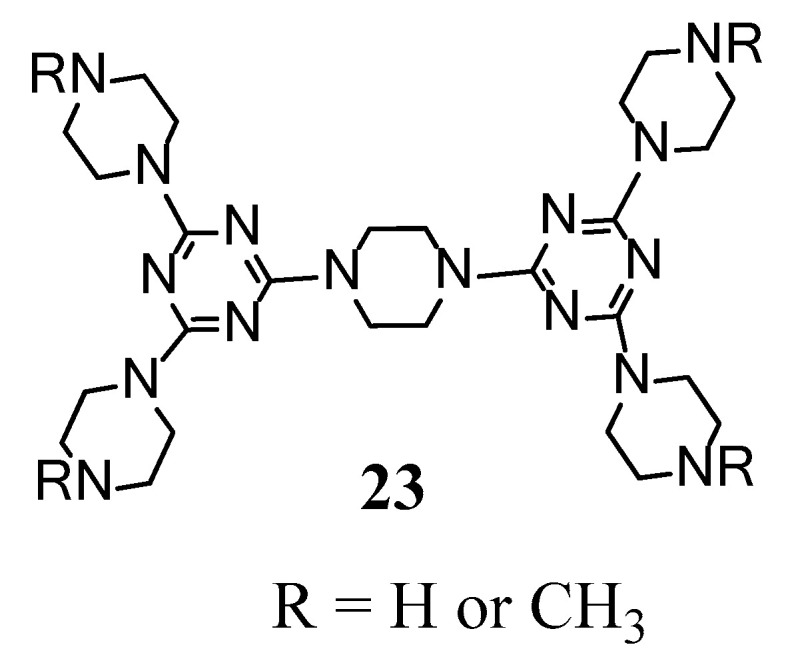
Structure of molecule (**23**).

Intermolecular H-bond interactions can also be applied to prepare 1,3,5-triamino-*s*-triazine-based dendrimers with the columnar phase. Figure 13 shows an example of a central core with a *C_2_* symmetry that can be efficiently prepared according to the literatures [62,63,64,65,66,67]. Incorporating special functional groups at the periphery of dendrons can give desired physical properties to the LC triazine-based dendritic mixtures. Although the photoconductivity of the resulting dendrimers from the mixtures of 2,4-diamino-6-dodecyl-1,3,4-triazine and the corresponding dendrons is not better than those of dendrons alone, this property may be improved if columnar phases are formed on the thermal treatment of the dendritic mixtures since these columnar mesogens show better long-range self-assembling ability. Compound **23** may be likely to form columnar mesophases when treated with proper acid-containing dendrons.

## Data Availability

The data presented in this study are available on request from the corresponding author.
